# New insight of square stepping exercise in immune fine-tuning for anticipating emerging pandemics

**DOI:** 10.1080/19768354.2024.2350157

**Published:** 2024-05-07

**Authors:** Hyo-Jeong Cha, Changwan Hong

**Affiliations:** a Department of Geriatric Rehabilitation, Youngsan University, Yangsan, Republic of Korea; b Department of Anatomy, Pusan National University School of Medicine, Yangsan, Republic of Korea

**Keywords:** Immune function, cognitive disorder, BDNF, stepping training, home-training

## Abstract

The COVID-19 pandemic has significantly impacted human life, posing serious physical and psychological threats, particularly to the elderly. While individuals of all ages are susceptible to contracting COVID-19, older people face a heightened risk of developing various diseases due to age-related immunophysiological changes and preexisting health conditions. The interplay between immune health and physical activity is believed to hold even greater significance during a pandemic. Recent findings from our research indicate that the intervention of square stepping exercise (SSE), characterized by a rhythmic and controlled stepping pattern, resulted in increased levels of Brain-Derived Neurotrophic Factor (BDNF) in the elderly. BDNF, known to influence not only nerve cells but also immune cells, suggests a potential link between SSE and immune system modulation. Consequently, this exercise regimen holds promise in counteracting age-related immunophysiological changes, fine-tuning immune responses, and mitigating the severity of potential new virus outcomes, such as ‘Disease X.’ This review aims to underscore the significance of integrating SSE as a home-based program, serving as a potent tool to enhance immune resilience, prepare for future potential pandemics, and empower older individuals during challenging times. Through the practice of SSE, older adults may strengthen their ability to navigate the challenges posed by pandemics and maintain a sense of control over their well-being.

## Background

As of 2023, owing to the SARS-CoV-2 outbreak during the end of 2019, there have been more than 774 million confirmed cases worldwide and a whopping 7 million deaths (WHO dashboard Dec, 2023). While there has been a gradual decline in viral transmission, recent developments have shown a sharp resurgence in the number of infected individuals, constituting a concerning trend (WHO dashboard Dec, 2023). Governments worldwide have responded with a range of containment measures, including quarantine protocols and protective strategies, such as physical distancing, self-isolation, mask mandates, and frequent hand washing, along with the promotion of telecommuting and the imposition of restrictions on private gatherings, business operations, and large-scale events (Baker et al. 2020; Chu et al. 2020). As a result, drastic changes have begun to appear in people's daily lives worldwide (El-Zoghby et al. 2020), including high COVID-19-related fears (Bauerle et al. 2020) and numerous psychological consequences such as depression (Alkhamees et al. 2020), increased sleep problems (Beck et al. 2021), and financial worries (Chakraborty and Chatterjee 2020).

Among these changes, pandemic psychosocial studies have reported that an individual's mental health is severely affected by COVID-19 and the related isolation (Brodeur et al. 2021). In fact, early studies on COVID-19 conducted in China (Ahmed et al. 2020; Xiao 2020) and studies done in Spain (Rodriguez-Rey et al. 2020; Munoz-Navarro et al. 2021) showed that the symptoms of anxiety, stress, and depression increased dramatically during the pandemic. In a study of 775 US adults, 55% recently reported that the pandemic had negative effects on mental health (Organization 2020). Other studies also found a higher rate of mental distress during periods of isolation (Sibley et al. 2020) and predicted that this was a consequence of frustration, boredom, depressed mood, and potential depression (Venkatesh and Edirappuli 2020). For the elderly, isolation and protection are inevitable and important because of the high risk of developing severe illness and death following COVID-19 (Schultze et al. 2020; Williamson et al. 2020). The side effects of such protective measures have been reported in a previous study, and this problem may worsen in combination with various geriatric diseases. Older adults already face issues such as loneliness, age discrimination, and excessive worry, and quarantine measures can exacerbate these problems (Griffin et al. 2020). Social isolation due to physical distancing can have adverse effects on mental health and daily living habits (Singh and Misra 2009), and elderly cognitive decline (Griffin et al. 2020) can be expected. Indeed, a study of older adults in New Zealand reported that such isolation could lead to higher levels of mental distress, lower mood, and depression compared to older control adults (Sibley et al. 2020; Venkatesh and Edirappuli 2020). Additionally, a recent UK study of people over 70 years old reported that the COVID-19 lockdown had a broad negative impact on the well-being, mood, stress, and memory domains (Docherty et al. 2021).

Among the various demographic groups affected, the elderly population has encountered formidable challenges spanning both the physical and psychological domains. Although the virus poses a threat to individuals of all ages, older adults are uniquely susceptible to severe outcomes owing to their altered immune responses resulting from physiological changes associated with aging and underlying health conditions. The pandemic and the intricate relationship between immune health and physical activity have accentuated the importance of exploring innovative approaches to safeguard the well-being of older individuals.

Therefore, square stepping exercise (SSE) has emerged as a promising avenue for enhancing immune resilience and overall well-being in the elderly population. This exercise regimen, characterized by its adaptable and accessible nature, holds potential as a proactive strategy to fortify the immune system and mitigate the detrimental effects of age-related immunophysiological alterations. The rhythmic and controlled stepping patterns intrinsic to SSE offer a platform for counteracting some immune deficiencies associated with aging, potentially reducing the severity of COVID-19 outcomes in older individuals. By investigating the integration of SSE as a home-based program, this study sought to underscore its significance as a tool for empowering and equipping older adults to navigate the challenges imposed by the pandemic. The incorporation of SSE into daily routines may offer older individuals a means to proactively manage their immune health, foster their overall well-being, and cultivate a sense of control in the face of adversity.

As we delve into the intricate interplay between immune health, physical activity, and the potential role of SSE, it is evident that a comprehensive understanding of these dynamics can shed light on effective strategies to enhance resilience in older adults during these challenging times. We aim to highlight the potential of SSE as a catalyst for immune enhancement, overall health improvement, and empowerment of older individuals, thereby contributing to collective efforts to mitigate the impact of the ongoing pandemic.

## Square stepping exercise

Regular exercise promotes physical and mental health in the elderly by reducing the risk factors for chronic diseases, providing social contact opportunities, and improving cognitive function related to mental health. The importance of regular exercise in old age is further emphasized by the quarantine caused by the COVID-19 pandemic. Although the benefits of exercise are widely known, many elderly people do not exercise regularly, and most elderly people with dementia cannot participate in regular exercise, even under non-pandemic circumstances. In addition, isolation due to the pandemic makes it more difficult for older adults to participate in regular exercise, which can cause serious healthcare problems. Therefore, the development of a home training program is required to solve healthcare problems and prevent dementia.

The SSE was initially developed by Shigematsu et al. to prevent falls and improve cognitive function (Shigematsu and Okura 2006). The SSE program consists of multiple directional step patterns, such as moving forward, backward, left, right, and obliquely, using a thin mat that is divided into 25-cm squares on a 100-cm wide and 250-cm long surface, with approximately 200 stepping patterns. SSE is aimed at improving both motor and cognitive function in the elderly (Shigematsu et al. 2008). In this regard, it would be of great help to instructors and users if there was a guide-book for SSE program that is clearly categorized and organized, but it is still difficult to find and access.

Executive functioning in daily life includes three key factors: inhibition, working memory, and cognitive flexibility. Optimal problem-solving is necessary when it is inappropriate or impossible to rely on instinct or intuition in situations that require attention (Diamond and Ling 2019). It has been reported that SSE stimulates these three core executive functions and improves aspects such as problem-solving ability, inference, and mental flexibility in young adults (Kawabata et al. 2021). In addition, SSE involves classical patterns of activity, such as walking and jumping, and has shown a positive effect on the motor and cognitive abilities of children (Dominguez-Munoz et al. 2021). Notably, although there is no direct scientific evidence, studies on the positive effects of exercise on immunity (Walsh and Oliver 2016) suggest that regular SSE participation could lead to beneficial changes in immune cell populations and function. The SSE program is an educational psychomotor intervention activity for the development of motor and cognitive abilities from children to the elderly, regardless of age. SSE enhances cognitive control, improving working memory and cognitive flexibility, and also functions required to plan and execute actions to achieve goals, including problem solving (Dominguez-Munoz et al. 2021; Kawabata et al. 2021).

For a healthy elderly life during a pandemic, it is necessary to develop a more efficient exercise program that can improve not only physical but also cognitive and immunological function. It is important to find a fused home-training program that includes not only functional development through simple movement, but also the improvement in immune function and cognitive ability. Since training programs that require specific locations and expensive tools should be excluded in terms of accessibility and ease of use by the elderly, SSE, which can be easily exercised on a thin fabric mat with low cost and no power required, is an optimal home training program that can simultaneously improve physical, cognitive, and immunological function in a pandemic environment.

### Square stepping exercise and physical function

Changes that increasingly appear with age include physical degeneration. In particular, lower limb muscle strength decreases by 30% from the 50s to 70s and even more rapidly after the 80s, increasing the vulnerability to falls (Lindle et al. 1997). Falls are the second leading cause of unintentional injury-related deaths worldwide, and age is a major risk factor. The elderly is at the highest risk of death or serious injury from falls, and the probability increases with age. An estimated 684,000 people worldwide die each year from falls, and more than 80% live in low- and middle-income countries. The World Health Organization (WHO) recommends gait, balance, and functional training as interventions to prevent falls in older adults. An aging study conducted in New Mexico reported that elderly people with sarcopenia were four times more likely to have a physical disability, two to three times more likely to have a physical balance disorder, and twice as likely to sustain a fall than healthy elderly people (Newman et al. 2003), emphasizing the importance of physical training to prevent falls.

In order to prevent a sudden fall, quick stepping ability and balance are crucial skills. Stepping exercises (SEs) should be accompanied by rapid movements and a high foot position, in addition to gait correction. Therefore, SEs are an essential component of a fall prevention program, and the focus of effective training should be on correct performance. It has been reported that SEs reduce falls by up to 50% in trained elderly individuals (Okubo et al. 2017). An observational study on the movement characteristics of elderly participants during a stepping game (exergaming) (Skjaeret-Maroni et al. 2016) and a fall prevention meta-analysis of 19,478 elderly people (Sherrington et al. 2017) showed fall prevention was promoted when difficulty was increased by adding cognitive factors or movements to the SE. SSE has additional training factors incorporated into the SE. SSE also includes principles of motor learning that can improve motor skills through practice, which is the basic theory of training. A recent meta-analysis reported that SSE has a significantly positive effect on fall risk, agility, and static and dynamic balance (Wang et al. 2021). A meta-analysis of the effects of SSE including studies from 2005 to 2016 reported that SSE was significantly more effective in improving perceived health and preventing falls and fear of falls than walking or a sedentary lifestyle (Fisseha et al. 2017).

### Square stepping exercise and cognitive function

Cognitive and physical exercises performed independently, regardless of cognitive impairment, can improve cognitive function (Kawabata et al. 2021). There are also reports that a combined intervention program with cognitive and physical exercises is beneficial for improving cognitive function (Karr et al. 2014). It has also been reported that a dual-or multitask exercise program is an effective intervention that reduces the risk of falls and improves cognition by minimizing physical and cognitive decline in elderly individuals (Giannouli et al. 2019). SSE consists of a variety of patterns, from easy to complex steps, to simultaneously increase motor and cognitive stimulation. SSE not only improves physical function through repetitive and continuous stepping and direction changes, paying attention to steps on the 25-cm square, but also stimulates neuronal cells by simultaneously performing memorized patterns for each difficulty level (Cha et al. 2022). A recent meta-analysis found that aerobic exercise helped improve cognitive function (Northey et al. 2018), and that low- and moderate-intensity exercises were particularly effective (Chang et al. 2012). According to the American College of Sports Medicine and Centers for Disease Control, low- and moderate-intensity exercise is defined as <3.0 and 3.0–6.0 MET, respectively (Haskell et al. 2007). SSE is reported to be as effective for the elderly as moderate-intensity exercise such as walking between 3.0 and 4.0 MET (Uchida et al. 2020). Sorte et al. reported that concentration, reasoning, memory, and overall cognitive function scores improved after 24-week exercise interventions, including SSE, in elderly people with subjective cognitive dissatisfaction, and that improvement in cognitive function scores was maintained even 28 weeks after SSE intervention (Boa Sorte Silva et al. 2018). A study that conducted an SSE program for elderly individuals with diabetes and self-reported cognitive complaints also reported a significant improvement in cognitive scores compared to the control group (Shellington et al. 2018).

Social interactions have a significant positive effect on cognitive stimuli. SSE can be effective for cognitive stimulation as it increases social interaction through physical contact and shared experiences, such as high fives, eye contact, communication, and observation of each other's exercise, while performing SSE in groups of approximately seven participants. However, because these interactive elements are excluded in a pandemic situation, the program should be developed considering these limitations.

### Square stepping exercise and immune function

As the world has grappled with the ongoing COVID-19 pandemic, understanding the potential immunomodulatory effects of SSE is particularly relevant for bolstering immunocompetence. The intricate relationship between exercise and the immune system is being explored with renewed vigor (Walsh and Oliver 2016; Llavero et al. 2021; Rosa-Neto et al. 2022).

Physical exercise has long been associated with a range of benefits to the immune system. Regular and moderate exercise have been shown to enhance immune function, improve immune surveillance, and reduce the risk of chronic inflammation (Simpson et al. 2020; de Souza Teixeira et al. 2021; Soares et al. 2021). These effects are attributed to exercise-induced changes in immune cell distribution, cytokine production, and immune signaling pathways (Millard et al. 2013; Moro-Garcia et al. 2014; Zhang et al. 2020). However, the extent and mechanisms of these effects can vary depending on the type, intensity, and duration of the exercise.

SSE stands out within the realm of exercise due to its rhythmic and repetitive stepping patterns. It combines aerobic exercise with controlled movement, engaging both the cardiovascular and musculoskeletal systems. This engagement leads to improved blood circulation, enhanced oxygen delivery to the tissues, and heightened lymphatic flow (Duggal et al. 2019). These physiological changes may have implications on immune cell trafficking, interactions, and overall function (Duggal et al. 2019).

Recent studies have revealed potential immunomodulatory effects of physical exercise. Regular exercise is associated with alterations in immune cell populations and functions (Gleeson et al. 2011; Duggal et al. 2019). For example, heightened natural killer cell activity, which is crucial for the elimination of virus-infected cells, was observed following acute exercise (Pedersen et al. 2016; Rumpf et al. 2021). One of the key ways in which exercise influences the immune system is by modulating cytokine production. Physical exercise is linked to the release of certain cytokines, including interleukin (IL)-6 and IL-10 (Docherty et al. 2022). These cytokines play an important role in immune regulation and control of inflammation (Jeong et al. 2023). IL-6, for instance, can have both pro- and anti-inflammatory effects, and its balance is crucial for immune homeostasis (Hunter and Jones 2015). The immune system's ability to mount effective responses to pathogens, including the SARS-CoV-2 virus responsible for COVID-19, is a critical factor in disease outcomes. As the elderly population is at a higher risk of severe COVID-19 outcomes, interventions that enhance immune function are of particular interest.

**Figure 1.**
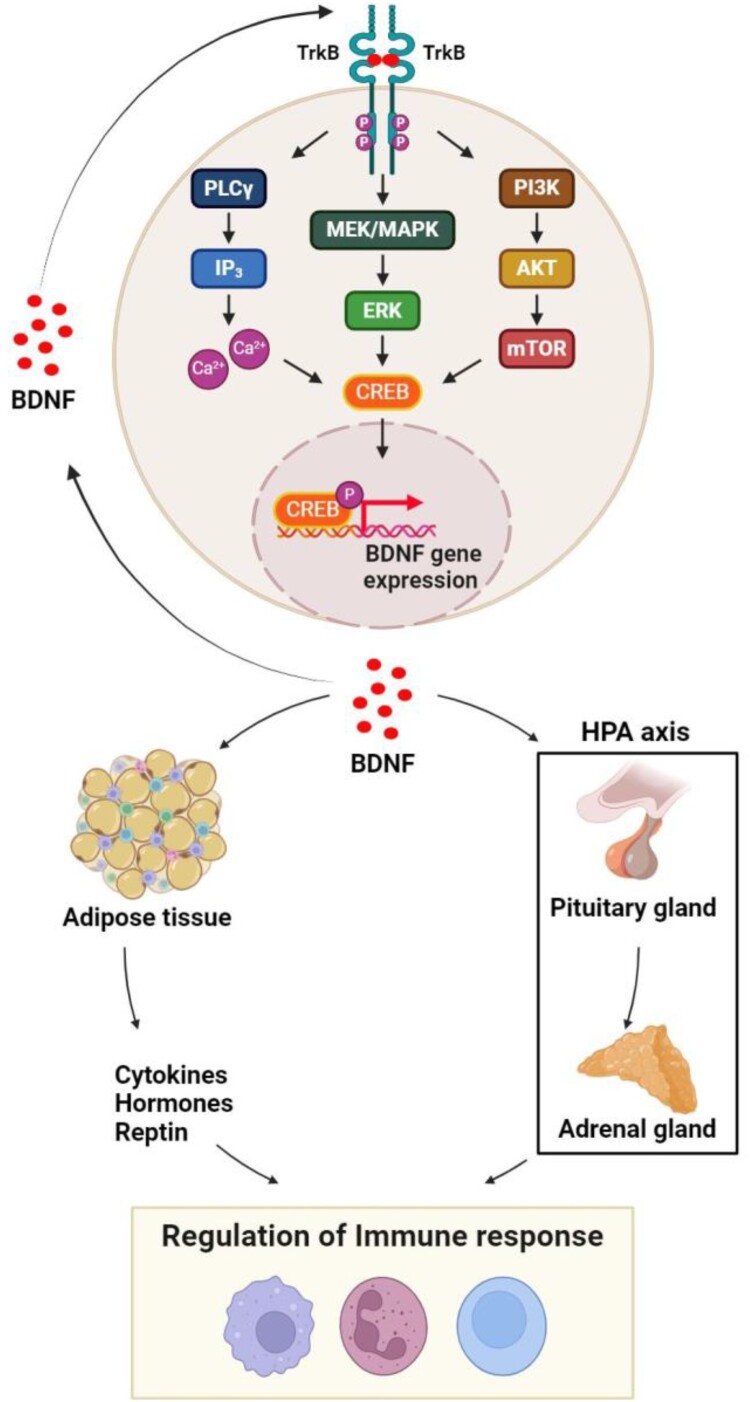
BDNF/TrkB/CREB signaling and immune regulatory pathway. The BDNF/TrkB signaling complex initiates the phosphorylation of the TrkB cytoplasmic domain, leading to the activation of the PLCγ, ERK, and AKT pathways. This activation subsequently triggers CREB phosphorylation. Once activated, CREB is translocated into the nucleus, promoting the transcription of the *BDNF* gene. BDNF, in turn, activates adipocytes and the pituitary-adrenal gland axis through the sympathetic nervous system, either in an autocrine or paracrine manner. These pathway activations are interconnected with immune cells, contributing to the regulation of immune responses.

Our recent study on the increase in Brain-Derived Neurotrophic Factor (BDNF) through SSE is of significant importance (Cha et al. 2022). According to this study, SSE has a positive impact on fall-related fitness and BDNF levels (Cha et al. 2022). BDNF plays a role in neurogenesis, synaptic plasticity, regulating neuronal excitability, and perception of stimuli (Brown et al. 2020). BDNF binds to tropomyosin receptor kinase B (TrKB), forming functional homodimeric receptor complexes (Reichardt 2006). Ligand biding leads to a trans-phosphorylation of the cytoplasmic domains and then the activation of cAMP response element-binding protein (CREB) via MEK/ mitogen-activated protein kinase (MAPK) pathway and phospholipase C gamma (PLCγ)/ Ca^2+^-calmodulin-dependent protein kinase (CAMK) and protein kinase C (PKC) pathway (Kowianski et al. 2018). BDNF signaling also induces activation of mammalian target of rapamycin (mTOR) through phosphatidylinositol 3-kinase (PI3 K)/Akt pathway (Kowianski et al. 2018). This induces the upregulation of BDNF expression and play a critical role of in neuroprotective functions (Kim et al. 2023). On the other hands, it has been suggested that the enhanced BDNF might regulate immune cells with leptin, cytokines, hormones produced by adipocytes or with activation of hypothalamic–pituitary–adrenal cascade (Dadkhah et al. 2024) (Figure 1).

BDNF expression decreases with aging, which can explain the decline in cognitive function in the elderly (Mattson et al. 2004). In a study involving elderly individuals without dementia, higher serum BDNF levels were associated with a reduced risk of dementia (Weinstein et al. 2014). Additionally, increasing BDNF levels through exercise has been reported to enhance cognitive function in Alzheimer's disease mouse models (Choi et al. 2018). However, BDNF not only affects cognitive function but is also increasingly recognized for its significant role in immune-related diseases. Its associations have been reported in chronic autoimmune inflammatory bowel diseases, including Crohn's disease and ulcerative colitis (Xu et al. 2020; Sochal et al. 2021). In an animal model of multiple sclerosis (MS; experimental autoimmune encephalomyelitis induction), BDNF has demonstrated symptom relief effects (Lee et al. 2012; Fletcher et al. 2018). BDNF also plays an important role in rheumatoid arthritis (RA), an autoimmune inflammatory disease. Joint cartilage cells in RA mice showed higher expression of BDNF and TrkB, which is associated to BDNF pathway, compared to WT mice (Grimsholm et al. 2008). Similarly, in humans, TrkB expression in the synovial fluid of RA patients was upregulated compared to healthy individuals (Barthel et al. 2009). Therefore, BDNF appears to play a crucial role in the pathophysiology of autoimmune diseases.

BDNF not only affects autoimmune diseases but also contributes to protective immune responses by regulating T cell responses. Research using an enriched environment (EE) eustress model that can induce social, physical, and cognitive stimulation, unlike standard environments (SE) commonly used in biomedical research, has shown that BDNF expression induced by EE inhibits tumor progression in melanoma and colon cancer models (Cao et al. 2010). Other research groups have confirmed similar anti-tumor efficacy in breast and pancreatic cancer models (Nachat-Kappes et al. 2012; Li et al. 2015). This anti-tumor effect is attributed to the EE-induced increase in BDNF and is manifested through enhanced function of CD8 cytotoxic T lymphocytes (CTL), which play a central role in anti-tumor immunity (Xiao et al. 2016). Another important cell that can induce anti-tumor and antiviral responses is Natural Killer (NK) cells. According to studies in the EE model, enhanced BDNF levels were reported to enhance NK cell maturation and cytotoxic function against melanoma and pancreatic adenocarcinoma (Cao et al. 2010; Meng et al. 2019; Bergin et al. 2021; Mansour et al. 2021).

**Figure 2.**
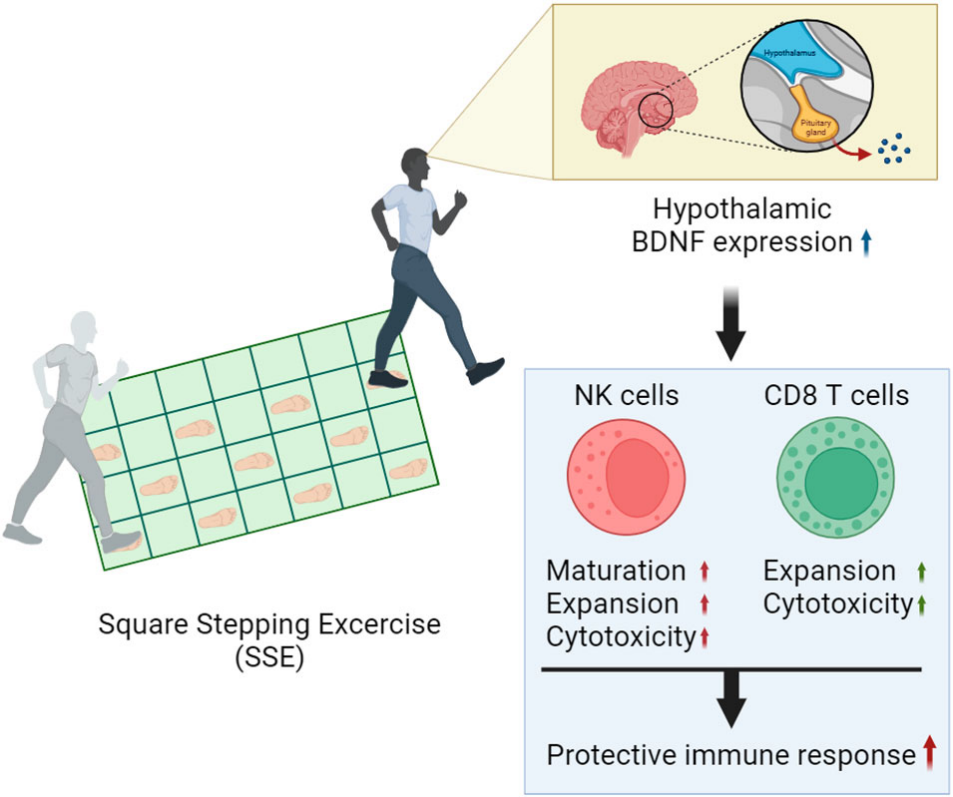
Relationship between SSE and protective immune responses. SSE stimulates the expression of BDNF in the hypothalamus. Elevated levels of BDNF are anticipated to enhance the functionality of NK and CD8 T cells, potentially resulting in improved protective immunity against viral and bacterial infections.

In addition to neuron cells, BDNF is also reported to be expressed in immune cells such as B and T cells as well as macrophages (Kerschensteiner et al. 1999; Kruse et al. 2007) and is particularly detected in inflammatory responses within the central nervous system (CNS), such as those associated with conditions like MS (Stadelmann et al. 2002). Furthermore, there is evidence suggesting that BDNF plays a role in the survival and activation of eosinophilic granulocytes (Nassenstein et al. 2003), while also being involved in the B and T cell development (Schuhmann et al. 2005; Berzi et al. 2008). As shown in our previous study (Cha et al. 2022), the increase in BDNF expression due to SSE is expected to contribute to the enhanced function of NK and CD8 T cells, and this functional improvement is likely to lead to enhanced protective immunity against viral and bacterial infections (Figure 2). Collectively, SSE can be considered a novel exercise therapy with the potential to influence the immune response and strengthen the immune system through BDNF, as mediator of neuro-immune interactions (Park and Jung 2022).

Although further studies are required to fully understand the effects of SSE on immune cell activation and responses, SSE's potential to improve immune cell activity, cytokine balance, and overall immune response could contribute to reducing the severity of future potential virus symptoms in this vulnerable group. SSE is an emerging exercise regimen with the potential to influence immune responses and enhance the immune system.

### Limitations

Despite the numerous advantages inherent to SSE, it is essential to delineate its limitations. First, there are concerns regarding the potential attenuation of SSE's impact due to the absence of direct social interaction. While the incorporation of measures that foster synergistic relational effects remains limited, the absence of interpersonal connections does not negate a program's efficacy. Second, a significant limitation lies in the feasibility of elderly individuals effectively utilizing virtual reality systems, which are a central component of SSE. The applicability of this technology across diverse national contexts, socioeconomic strata, and age cohorts is inherently limited. Consequently, it is imperative to formulate and implement alternative approaches in line with the prevailing economic and sociocultural context. The third limitation is linked to the need for continued participation in order to achieve meaningful effects. In this regard, the design and implementation of strategies aimed at bolstering adhesion are paramount. In particular, when applied in a domestic setting, a comprehensive framework is indispensable for optimizing the sustained efficacy of SSE initiatives. In summary, acknowledging the limitations of SSE engenders an informed perspective that views these challenges as potential avenues for targeted interventions and refinement. By addressing concerns related to social interaction dynamics, technology inclusivity, and maintaining motivation, the latent potential of SSE programs can be harnessed to improve well-being across a diverse spectrum of demographic categories.

## Practical applications

The expected effects and application plans of the SSE program for the elderly during the COVID-19 pandemic can be broadly summarized as follows:

1. Exercises that require specific equipment or locations are not currently suitable in quarantine environments because of the pandemic. Therefore, SSE, which only requires the use of a square-printed fabric mat, constitutes an ideal exercise modality. It is recommended that the physical and cognitive functions of the elderly population are enhanced.
2. Although the importance of strength training increases with age, it is often not practiced due to concerns regarding injuries. However, SSE is a moderate-intensity exercise similar to walking, and can be safely used as an intervention for the elderly.
3. Although exercise programs for the elderly vary according to age, the strength levels among individuals of the same age are significantly different. No exercise program considers individual strength deviations. However, as SSE does not require special exercise skills or strength, it attenuates these individual differences.
4. Since SSE is conducted with several participants, it has advantages in terms of cultivating social bonds and relationships, but the isolation imposed by the pandemic negates these benefits. Therefore, it is necessary to overcome such limitations by introducing virtual reality technology, such as the Metaverse, which has recently been developed.

## Conclusions

By introducing a comprehensive SSE program tailored for home-based practice, this study endeavors to offer a practical solution to the challenges that older individuals face in maintaining their health during the pandemic. The program encompasses not only physical exercise, but also cognitive engagement, acknowledging the multifaceted nature of health in aging. This initiative holds promise as a means of empowering older adults and allowing them to actively contribute to their well-being while navigating the complexities of the pandemic. The interplay between exercise and the immune system is a dynamic and complex area of study that is gaining significance in the context of the COVID-19 pandemic. SSE, with its rhythmic and controlled stepping patterns, offers a potential avenue for enhancing immunocompetence. The growing body of research exploring the effects of SSE on immune cell activity, cytokine signaling, and overall immune responses opens exciting possibilities for using exercise as a proactive strategy to support immune health and mitigate the impact of infectious diseases such as ‘Disease X’. Further research is required to elucidate the precise mechanisms by which SSE influences the immune system and to optimize its incorporation into broader health strategies.

## List of abbreviations

COVID-19, coronavirus disease; IL, interleukin; SSE, square stepping exercise; SE, stepping exercise.

## Conflicts of interest

The authors declare that they have no competing interests.
